# Molecular Docking and Pharmacokinetics Prediction of Piperine and Capsaicin as Human Pancreatic Lipase Inhibitors: An In Silico Study

**DOI:** 10.7759/cureus.67870

**Published:** 2024-08-26

**Authors:** Harismitha S, Neha Deora, Ameer Khusro

**Affiliations:** 1 Department of General Medicine, Saveetha Medical College and Hospitals, Saveetha Institute of Medical and Technical Sciences, Saveetha University, Chennai, IND; 2 Department of Research Analytics, Saveetha Dental College and Hospitals, Saveetha Institute of Medical and Technical Sciences, Saveetha University, Chennai, IND

**Keywords:** natural products, dietary fat, obesity, piperine, pancreatic lipase, capsaicin

## Abstract

Introduction

Obesity is a complex multifaceted disease, characterized by excessive body fat accumulation. It is a major public health concern globally, affecting individuals of all ages, genders, and socioeconomic backgrounds. Lipase, a key enzyme involved in lipid metabolism, plays a crucial role in the hydrolysis of dietary fats. Pancreatic lipase performs hydrolysis of nearly 50%-70% of total dietary fats. Thus, inhibition of pancreatic lipase is recognized as one of the strategies for managing obesity.

Aim

To predict the effect of phytocompounds from pepper as pancreatic lipase inhibitors using computational approaches.

Methodology

The drug-likeness and pharmacokinetic properties of compounds were evaluated using Lipinski rule of five and absorption, distribution, metabolism, and excretion (ADME) analysis, respectively. The drug score value was computed using Molinspiration, while the lipase inhibitor potential of ligands was evaluated using prediction of activity spectra for substances. Molecular docking was carried out to evaluate the stability and ligand binding affinity.

Results

Computational approaches identified both piperine and capsaicin as potential candidates, exhibiting favorable affinities with binding energy values of -9.9 and -7.7 kcal/mol, respectively. Both piperine and capsaicin interacted with Ser-152 and His-263, demonstrating their binding at the substrate binding site.

Conclusions

Findings provide insights into the underlying anti-obesity potential of these bioactive compounds from pepper and support further experimental investigations for obesity treatment.

## Introduction

Obesity, defined by excess adiposity and increased ectopic accumulation, has been linked to the progression of a wide range of chronic disorders [1]. It is considered a global health problem by the World Health Organization (WHO), with 2.5 billion adults being overweight, of these 890 million were living with obesity in 2022 [2]. It is widely recognized to play an important role in metabolic disorders and the syndrome of energy equilibrium. Obesity is becoming more prevalent due to the disruptions in lipid homeostasis caused by genetic, environmental, and lifestyle factors. Sedentary lifestyles and the easy availability of high-calorie foods, resulting in an energy imbalance in genetically predisposed individuals, are contributing factors to the startling rise in obesity [3].

The majority of dietary fat, predominantly composed of mixed triglycerides, is not absorbed by the intestine directly unless the fat has been subjected to the action of lipases [1]. Thus, one of the most researched targets among obesity treatments is the regulation of energy use and storage in adipocytes, as well as the inhibition of digestion and intestinal absorption of dietary fat by lipases. Among various lipases, pancreatic lipase hydrolyses nearly 50-70% of total dietary fats [4]. Inhibition of fat hydrolysis decreases the utilization of ingested lipids; therefore, inhibition of lipases reduces the absorption of fat. Thus, pancreatic lipase inhibition is an interesting advancement toward the development of potential therapeutic agents for obesity management and associated complications [5]. Orlistat is a well-known selective inhibitor of pancreatic lipase, but due to the unwanted gastrointestinal side effects associated with it and long-term therapy, there is a need to search for alternatives that are cost-effective and can offer therapeutic potential with reduced side effects [6].

Plants-based products are storehouses of a variety of phytocomponents or bioactive molecules such as phenolics, flavonoids, terpenes, and sterols. Thus, the identification of phytocomponents, or bioactive molecules, from plants provides a framework and an opportunity for the development of pancreatic lipase inhibitors that could eventually be developed into anti-obesity drugs [7]. Piperine and capsaicin are bioactive compounds found abundantly in black (*Piper nigrum*) and chili (*Capsicum annuum*) pepper, respectively. They exhibit a variety of beneficial biological activities, including, antimicrobial, anti-inflammatory, gastro-protective, antidepressant, and antioxidant [8,9].

Piperine is a nitrogen-containing alkaloid abundantly present in black, green, and white pepper [10]. It has received considerable attention in the last two decades due to its protective and therapeutic effects against numerous diseases including, diabetes, hypertension, cancer, cardiovascular, and reproductive diseases, and can modulate various signaling pathways [11]. Also, it enhances nutrient and drug bioavailability by inhibiting metabolic enzymes that catalyze the biotransformation of nutrients [12]. It has been reported to have anti-obesity potential, and these effects are due to its ability to reduce fatty acid absorption and increase energy expenditure along with enhancement of intestinal barrier [13].

Capsaicin is a bioactive compound abundantly found in chili pepper and has been established to have numerous biological activities [14]. Further, dietary red pepper has been reported to suppress energy intake and modify macronutrient intake through appetite and satiety regulation [15]. Capsaicin has also been reported to promote weight loss and improve metabolic health by increasing energy expenditure and lipid oxidation in numerous clinical trials [16,17]. Further, the administration of capsaicin for eight weeks increased brown adipose tissue (BAT) activity and thermogenesis in healthy individuals [18]. Also, capsaicin has been demonstrated to inhibit adipogenesis in 3T31 preadipocytes by alleviating the expression of PPARγ, and leptin [19]. However, their inhibitory potential against pancreatic lipase using computational studies is scanty. In the present study, we used computational approaches to investigate the inhibitory potential of piperine and capsaicin against pancreatic lipase. The discovery of new drugs using *in silico* approaches simplifies the overall process since it involves several stages and workflows. The insights gained from this research will help understand its mechanism and can be beneficial in developing natural treatments for obesity and related metabolic disorders.

## Materials and methods

Phytoconstituents of interest

**Figure 1:**
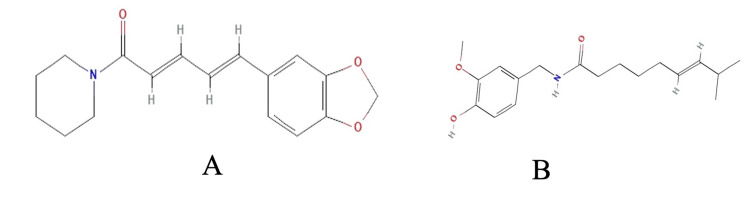
Structures of (A) piperine and (B) capsaicin. Structures of piperine and capsaicin were obtained from PubChem.

Structures of the phyto-components were retrieved from PubChem in the structure data file (SDF). The structure of piperine and capsaicin is illustrated in Figures 1A-1B, respectively. Orlistat was used as a standard inhibitor for lipase.

Target receptor

For docking studies, the three-dimensional crystal structure of pancreatic lipase (PDB: 1LPB), used as the protein receptor, was retrieved from RCSB PDB. The complexes bound to the receptor, such as non-essential water molecules and any inhibitors, were removed while docking.

Molecular docking

The molecular docking performed in the present study is blind. Before docking, the protein receptor was assigned with bond order, missing atoms, and hydrogen atoms using Auto dock tools (ADT). Docking calculations were performed with Auto Dock Vina. In blind docking, the whole protein fits inside the grid box and uses a rectangular box for the binding site. The box center was defined and the docking box was displayed using ADT. Following a docking experiment on Auto dock, the 5 best binding positions per docking run were analyzed using their docking score (docking score represents the minimum potential energy where protein and ligand come together and mathematically it is Score = S [target-ligand] + S [ligand] [20]. The preferred conformations were the ones with the lowest binding energy within the active site. Finally, the docking results generated were directly loaded into Discovery Studio visualizer, v 2016 [21]. Orlistat, an inhibitor of lipase was docked as a positive control

Lipinski’s rule of five

Lipinski’s rule of five was implied to determine the drug-likeness of all the ligands. This rule not only illustrates the durability but also demonstrates the molecular weight, log P, number of hydrogen bond acceptors, number of hydrogen bond donors, and molar refractivity of the drug candidate.

Absorption distribution metabolism excretion properties

Ligands satisfying Lipinski’s rule of five were subjected to ADME (absorption, distribution, metabolism, and excretion) properties analysis using the Swiss ADME tool of the Swiss Institute of Bioinformatics (SwissADME). The canonical SMILES were retrieved from PubChem and evaluated by the Swiss ADME tool. Properties, such as water solubility (Log mol/L), lipophilicity (Log Po/w), gastrointestinal (GI) absorption, blood-brain barrier (BBB) permeant, and P-gp substrate were estimated using this tool. The Swiss ADME tool is based on the principle of vector machine algorithm that can easily analyze data sets of known inhibitor/non-inhibitor, as well as substrate/non-substrate [22]. These phytocomponents were selected further for molecular docking analysis.

Bioactivity score prediction

The overall potential of a compound to be a drug candidate is indicated by its bioactivity score value. It is a measure of their ability to interact with specific receptors and predict whether they can act as a drug or have other pharmacological effects. Mol Inspiration is a computer-based program (Mol Inspiration Cheminformatics) that uses Bayesian statistics to compare ligand structures identify features typical of active molecules and predict the bioactivity score of compounds against common human receptors [23].

*In silico* prediction using Prediction of Activity Spectra for Substances

Prediction of Activity Spectra for Substances (PASS), a computer-based program, was used to screen the lipase inhibitory potential of piperine and capsaicin. Based on a structure-activity relationship with a known chemical entity, the software predicts the biological activities of chemical structures, including phytocompounds. Along with the desired pharmacological effect, it also forecasts the molecular mechanism of action and the unfavorable side effects. The compound is compared with a training set containing over 205,000 chemicals that display over 3750 different types of biological activity. The estimated activity is expressed in terms of probable activity (Pa) and probable inactivity (Pi). For a certain pharmacological effect, compounds with Pa greater than Pi are taken into consideration [24].

## Results

Molecular docking

**Figure 2:**
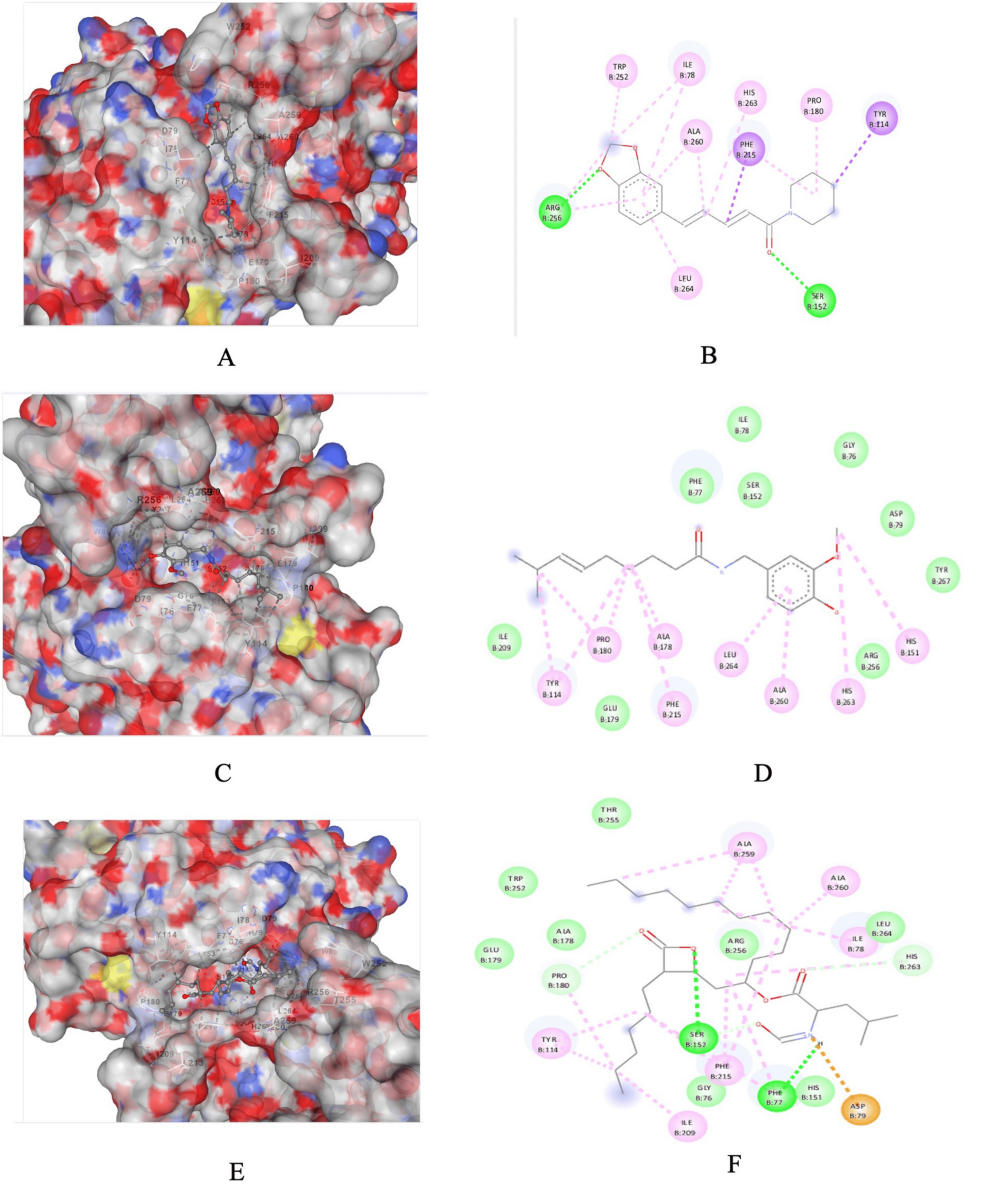
Crystal structure of pancreatic lipase and binding of bioactive compounds. (A) Surface representation of the overall three-dimensional structure of lipase and binding of piperine, (B) proposed binding amino acid and type of interaction are shown in different colors, (C) surface representation of the overall three-dimensional structure of lipase and binding of capsaicin, (D) proposed binding amino acid and type of interaction are shown in different colors, (E) surface representation of the overall three-dimensional structure of lipase and binding of orlistat, (F) proposed binding amino acid and type of interaction are shown in different colors. These data are generated by researchers.

The 3D and 2D representations of the interaction between the target proteins, the phytocompounds, and orlistat (standard drug) are presented in Figures 2A-2F. Piperine exhibited the lowest binding energy (-9.9 kcal/mol), followed by capsaicin ( -7.7 kcal/mol). The free binding energy of piperine and capsaicin was found to be lower than orlistat (-6.5 kcal/mol) and the binding was at the active site of the protein. The lowest binding energy shows the highest binding affinity between receptor and ligand. The predicted pose and the interaction between pancreatic lipase and piperine show that the interactions are stabilized by hydrogen bonds formed by Ser 152 and Arg 256; and a pi-pi stacked bond with Tyr 114 and Phe 215. The interaction between pancreatic lipase and capsaicin shows that interactions are stabilized by a carbon-hydrogen bond formed by Ser 152, Arg 256, Phe 77, ILE 78, Gly 76, Asp 79, and Tyr 267; and pi-alkyl bond with Tyr 144, Pro 180, Leu 264, His 363, and His 151. 

Drug likeliness and ADME/pharmacokinetics

The drug-likeness properties of piperine and capsaicin predicted using Lipinski’s rule of five and ADME properties are presented in Table 1. According to this rule, a ligand will likely be orally active, if (1) molecular weight < 500 Da, (2) the calculated octanol/water partition coefficient (log P) < 5, (3) hydrogen bond donors (HBD), notably OH and NH groups <5, (4) hydrogen bond acceptors (HBA), notably N and O <10 and, (5) molar refractivity ranges from 40 to 130 [25]. Both piperine and capsaicin have three hydrogen bond acceptors, and hydrogen bond donors are 0 and 2 for piperine and capsaicin, respectively. The molecular mass for both phytocompounds is less than 500 Da and the molar refractivity for both piperine and capsaicin is less than 100. Results revealed approval of drug-likeness attributes of both piperine and capsaicin. Both are non-toxic and have high intestinal absorption. Topological polar surface area (TPSA) was less than 100 Å for both piperine and capsaicin, which features the oral drug potential of both phytocompounds. The bioavailability score for both compounds was 55%, and they demonstrated hydrophilic and lipophilic properties. Piperine and capsaicin showed better solubility than orlistat with LogS values of -3.74 and -3.53, and -7.6, respectively. Both phytocompounds are not substrates for P-gp, thus further confirming their drug-likeness attributes.

**Table 1:** Pharmacokinetics properties of ligands.

Properties	Piperine	Capsaicin	Orlistat
PubChem Id	638024	1548943	3034010
Molecular weight	285.34 g/mol	305.41 g/mol	495.7 g/mol
No. of rotatable bonds	4	10	24
No. of hydrogen bond acceptors	3	3	5
No. of hydrogen bond donors	0	2	1
Molar refractivity	78.08	91.47	159.83
Topological polar surface area	38.77	58.56	81.7 Å
ESOL (Log S)	-3.74	-3.53	-7.6
Solubility	Soluble	Soluble	Poorly soluble
Bioavailability score	0.55	0.55	0.55
Lipinski	0 violation	0 violation	1 violation
Log P _o/w _(iLOGP)	2.869	3.71	6.79
Gastrointestinal absorption	High	High	Low
Drug likeliness	Yes	Yes	Yes
P-glycoprotein substrate	No	No	Yes

Bioactivity score prediction

The bioactivity of compounds describes their beneficial effects on living beings. The bioactivity score was calculated for different parameters: activity on the G protein-coupled receptor ligand and nuclear receptor ligand, modulation of ion channel, inhibition of kinases, and proteases, and activity of other enzymes. The bioactivity score is presented in Table 2. The bioactivity score of piperine and the standard drug, orlistat, was > 0, whereas capsaicin had a bioactivity score between -5.0 and 0.

**Table 2:** Bioactivity score of ligands. GPCR, G-protein-coupled receptor

Bioactivity score	Piperine	Capsaicin	Orlistat
GPCR Ligand	0.10	-0.18	0.35
Ion channel modulator	-0.09	-0.10	0.06
Kinase inhibitor	-0.21	-0.28	-0.19
Nuclear receptor ligand	0.09	0.12	0.09
Protease inhibitor	0.12	-0.06	0.45
Enzyme inhibitor	0.07	0.00	0.43

PASS calculations for lipase inhibitor

The biological activity spectra of piperine and capsaicin were determined using an online version of PASS software. The results obtained are presented in Table 3. Capsaicin and orlistat were demonstrated to have lipase inhibitory activity with Pa of 0.055 and 0.396, respectively. Further, capsaicin was shown to have lipoprotein lipase inhibitory activity with Pa of 0.408.

**Table 3:** Prediction of activity spectra for substances - probability of compounds being active (Pa) or inactive (Pi). Pa, prediction of being active; Pi, prediction of being inactive

Activity predictions	Piperine		Capsaicin		Orlistat	
Activity	Pa	Pi	Pa	Pi	Pa	Pi
Lipoprotein lipase inhibitor	-	-	0.408	0.070	0.229	0.170
Anti-inflammatory	0.344	0.127	0.266	0.196	-	-
Phospholipase inhibitor	-	-	0.116	0.081	0.138	0.059
Lipase inhibitor	-	-	0.055	0.048	0.396	0.002

## Discussion

In recent years, one of the most important advances in drug development has been the implementation of *in silico* methodologies. Molecular docking has become an increasingly popular tool for the discovery of new drugs since it is an inexpensive screening technique that explores the behavior of small molecules at the binding site of a target protein. The most significant and sensitive binding affinity values are those that represent the largest magnitude negative number (highest binding affinity or lowest binding energy) that represents the most favorable conformation of the complex formed when the involved ligand efficiently binds with the active pockets of the target [26]. In the present study, piperine showed maximum binding interaction with lipase having a binding energy of -9.9 kcal/mol. These docking results are in agreement with the anti-obesity potential of piperine as reported earlier by Wang et al. [13]. According to the study, weight reducing effects of piperine are due to its ability to downregulate genes related to fatty acid absorption, fatty acid-binding protein 2, and a cluster of differentiation 36, along with enhancement of intestinal barrier function by downregulating jejunal tumor necrosis factor-α and lipopolysaccharide-induced damage on intestinal cell proliferation. Further, piperine regulates obesity-induced dyslipidemia in high-fat diet rats and significantly reduces body weight [27]. The present study reports its lipase inhibitory potential, further confirming its role in reducing the ingestion of dietary fats.

The binding energy for capsaicin against lipase was found to be -7.7 kcal/mol. These results were by earlier *in vitro* studies, reporting that capsaicin had a moderate affinity towards pancreatic lipase and molecular docking studies had shown that capsaicin revealed a strong binding affinity towards triacyl glycerol lipase with a binding energy of -7.7 kcal/mol [28]. The free binding energy of piperine and capsaicin was found to be lower than that of orlistat -6.5 kcal/mol, suggesting that both phytocompounds showed promising results for lipase inhibitory activity as compared to orlistat. The N-terminal domain of PL contains the catalytic triad with amino acids such as Ser-152, Asp-176, and His-263. In particular, serine is considered important for the catalytic activity of the PL [29]. Piperine and capsaicin interacted with both Ser-152 and His-263, demonstrating that both the phytocompounds hindered the substrate binding site, with better binding affinity for lipase than orlistat.

In this context, both phytocompounds revealed acceptable drug-likeness properties and ADME parameters. Log P is related to the lipophilicity of compounds. It is a measure of how well the drug dissolves in lipids or nonpolar solvents and is useful for the prediction of the absorption of drugs across the intestinal epithelium. Lipophilicity ranging from 0 to 5 is usually considered optimal for drug development [22]. In the present study, both piperine and capsaicin had Log P < 5, implying they would be well absorbed through the membrane into circulation (good bioavailability). Also, both phytocompounds were highly soluble, with a Log S value of less than -5, an indication that these compounds could achieve good bioavailability when administered orally. The number of rotatable bonds is a simple topological parameter that measures the flexibility of molecules and is a good descriptor of the oral bioavailability of drugs [25]. Further, TPSA is closely associated with the hydrogen bonding potential of a compound and good indicator of the oral bioavailability of the drug molecule. TPSA values for compounds values > 140 Å show poor intestinal absorption [30]. Both piperine and capsaicin had several rotatable bonds < 10 and TPSA less than 100 Å, featuring their molecular flexibility and oral drug potential.

The bioactivity score is calculated for active drugs towards parameters like ion channel modulators, kinase inhibitors, GPCR ligands, nuclear receptor ligands, protease inhibitors, and other enzyme inhibitors. The score allows efficient separation of active and inactive molecules [31]. The bioactivity score of a compound determines its potential to interact with its pharmacological targets. The bioactivity score of any compound can be predicted as active (bioactivity score > 0), moderately active (bioactivity score lies between −0.50 and 0.00), and inactive (bioactivity score < - 0.50) [32]. The bioactivity score of both piperine and standard drug orlistat was more than zero, suggesting considerable biological activities, whereas capsaicin had a bioactivity score between -0.50 and 0, indicating moderate biological activity. The results revealed that the physiological actions of these compounds might involve multiple mechanisms and could be due to the interactions with GPCR ligands, nuclear receptor ligands, and inhibitors of proteases and other enzymes. Also, the present study highlighted the utility of PASS, a computer-aided program to make a clear comparison of predicted and observed pharmacological properties of piperine and capsaicin. Calculations using PASS software have been used to confirm and correlate the biological activities of compounds [33]. The PASS analysis predicted the biological activity of capsaicin and orlistat for lipase with Pa values of 0.055 and 0.396, respectively. Both capsaicin and orlistat were shown to have lipoprotein lipase and phospholipase inhibitory activity, whereas piperine did not show any biological activity for lipases. The results obtained from bioactivity score prediction and molecular docking were in accordance, where piperine showed a better binding affinity for lipase with a binding energy of -9.9 kcal/mol and a bioactivity score of > 0.

Numerous strategies have been investigated to prevent and treat the increased prevalence of obesity and associated disorders. One such approach is the inhibition of pancreatic lipase to reduce fat hydrolysis and the utilization of ingested lipids [34]. Because natural products are an exemplary source of bioactive components (s) with a wide range of therapeutic benefits, there has been a continuous quest for lipase inhibitors from these sources. Piperine and capsaicin investigated in the present study are the bioactive compounds abundantly present in black and red chili peppers, respectively [10,14]. They have received considerable attention in the past two decades due to their protective and therapeutic effects against numerous diseases.

It can be noted that even though PASS analysis predicted the probability of capsaicin being a lipase inhibitor based on its structure, it is crucial to remember that PASS analysis only provides a probabilistic prediction and not the exact value. Thus, even while it’s a valuable tool, it does not ensure precise outcomes for specific compounds. This study confirms their anti-obesity potential using computational approaches through inhibition of pancreatic lipase and paves the way for future investigations towards the development of anti-obesity drugs. The limitation of the present study is the lack of a molecular dynamic simulation that reproduces the behavior of molecular systems and provides dynamic insights into protein-ligand interactions. Also, molecular simulation studies give the root mean square deviation value, which is a measure of the structural stability of the protein-ligand complex.

## Conclusions

In a nutshell, piperine and capsaicin favored the drug-likeness and ADME properties criteria. The bioactivity scores of piperine (more than 0) and capsaicin (-5.0 to 0) represented these compounds as *active* and *moderately active*, respectively. Molecular docking analyses predicted comparatively higher binding interaction of piperine (-9.9 kcal/mol) and capsaicin (-7.7 kcal/mol) than orlistat (-6.5 kcal/mol) against lipase receptors. In addition, as per PASS calculations, capsaicin exhibited prominent lipase inhibitory activities. Thus, this *in silico* study provides insights into the underlying mechanism for the anti-obesity potential of piperine and capsaicin and makes them valuable agents for further experimental investigations in the development of natural treatments for obesity and its associated metabolic disorders.
